# Progress in Fevers

**Published:** 1904-03-05

**Authors:** 


### Progress in Fevers.

(*Continued from page* 392.)

**Plague.**—By the end of 1902 it was hoped that plague had been stamped out in Manila, but it has reappeared again probably by importation from the endemic plague centres of China.[43] During the four years ending April 1903, something like 872 cases have occurred, with 724 deaths or a mortality of 83·0 per cent. The disease shows a decided racial preference. The ratio of attack per 1,000 of the calculated population during this period works out at 9·18 per 1,000 for the Chinese, 1·5 per 1,000 for the Filipinos, and 0·4 per 1,000 for the white population The case mortalities for the same period were Chinese 82·2 per cent., Filipinos 84·1 per cent., and among the few American cases 50 per cent. The lack of vital resistance to the disease shown by the dark-skinned races was well seen in the epidemic of plague in Australia in 1902, when the death-rate among the Chinese and the white population attacked was respectively 80 per cent. and 30 per cent. The greater prevalence of the disease among the Chinese may in part depend on racial susceptibility, but still more is it due to their insanitary and unclean personal habits, and to their herding together in dark, damp, overcrowded dwellings. In Manila many of them are congregated in massive old Spanish buildings successfully built to withstand time and earthquakes, and of such strength that nothing short of a charge of dynamite would annihilate them. These buildings are hopelessly dark, damp, and ill ventilated, they have few sanitary conveniences, and their mud floors are charged with the filth of three centuries. The Filipinos live in slighter dwellings less calculated to harbour rats and plague bacilli, but the lower-class Filipinos, as well as the Chinese, generally go barefoot, and consequently are very liable to get abrasions on the lower extremities through which plague bacilli find an easy entrance. In accordance with this view is the clinical fact that nearly all the cases of plague among the Chinese are of the bubonic form, and that the glands involved are almost always the inguinal or femoral groups, rarely those of the neck or axilla. The highly contagious primary pneumonic form, rare among the masses of the people, is the common type when whites or better class natives are attacked. The disease seems to be readily propagated from rat to rat and from rat to man, but rarely from man to man. Sporadic cases are not followed by local outbreaks and the incidence among quarantined plague contacts is very slight. The Board of Health are taking vigorous measures to exterminate rats. About 100 official ratcatchers are employed who receive a salary and also a bonus on each rat caught. They put out much poison and handle about 2,000 traps. A bounty on rats is also offered to the general public. Vigorous measures are also taken to destroy these animals on the vessels entering the Pasig River and to prevent them getting on shore from the ships. All the rats killed on ship and shore are labelled with the address where found and examined for plague bacilli. Special care is taken to exterminate them in an infected house and its immediate neighbourhood and the dwellings are disinfected. Contacts are removed, disinfected and inoculated with Shiga's antiseptic serum. They are then allowed to return after their houses have been thoroughly disinfected, but are medically inspected daily for nine days. Houses in which plague rats have been captured are similarly disinfected and treated as regards the extermination of the vermin. Later reports[44] tend to show the great value of prophylactic inoculation carried out among the Chinese in Manila. Experience here would indicate that inoculation with antiseptic prophylactic virus, repeated in ten days, is as certain a preventive of plague as vaccination is of small-pox. The reaction accompanying inoculation is fortunately only slight and is not productive of much bodily discomfort, hence there has been no very serious opposition to its employment among the Chinese population. As a result of the experiments of W. J. Simpson[45] in Hong Kong it has been shown that monkeys, pigs, calves, sheep, hens, geese, ducks, turkeys and pigeons can contract plague by feeding on plague-infected material. Since these observations were made plague infected poultry have been discovered in the western market of Hong Kong. This discovery of the possibility of food being infected with plague may lead to important modifications of the view that plague is mainly spread by inoculation. Probably man would not escape infection through the alimentary canal, since monkeys and other animals are readily infected in this way. This possible mode of infection is a question of great importance for the East, where food is often carelessly stored and very improperly cooked, and where the association with the lower animals is very intimate. The disease in animals who have been fed on infected material appears to be of a chronic and ill-defined type, a fact which seriously adds to the danger. In India plague is unfortunately becoming more widespread every year.[46] At the same time experience has shown the necessity for decreasing rather than increasing the severity of measures undertaken for its repression, owing to the ignorance and prejudices of the dense populations. In April last over 30,000 deaths a week from plague were being recorded, the Punjaub accounting for about half this number. Compulsory measures for isolation, evacuation, and disinfection have been abandoned generally as worse than useless. In their stead facilities for voluntary inoculation and disinfection are being offered with good results. A great scheme last year to carry out 10,000,000 voluntary inoculations in the Punjaub in six months was most unfortunately nipped in the bud by an outbreak of tetanus after 200,000 had been inoculated. In Allahabad the system of *laissez faire* has worked well. Advice and assistance have been freely offered, but no attempt has been made to enforce any protective or remedial measures. This has promoted good feeling between the authorities and the people, and the ravages of the disease appear to have been at least checked by certain measures adopted with the hearty goodwill of the people. Stricken houses disinfected during 1901–2 returned many less cases of plague during 1902–3 than did the houses which escaped disinfection. In some places, taught by experience, the people are learning the value of voluntary evacuation of their villages on the first appearance of plague or even of a sick rat. Recently several limited outbreaks of plague have been reported in the Lebanon [47] near the Turco-Persian frontier. In one of the infected villages dead rats were observed in the village shops. J. M. Atkinson,[48] principal civil medical officer of Hong Kong, gives some details of cases of plague successfully treated by large doses of carbolic acid. Of the six cases recorded five were English and one Japanese. Twelve grains of carbolic acid were given in mixture every two hours. Other measures—stimulants, local treatment of buboes and tonics, strychnine, digitalis and others, were employed also. None of the patients appear to have suffered from carboluria. One severe septic case received over 2,500 grains before the blood was free from plague bacilli.

**Small-pox.**—J. E. Sandilands [49] has made an analysis of the vaccination statistics of the Metropolitan Asylums Board for 1901 and 1902, from which certain lessons of the London small-pox epidemic can be deduced. The cases dealt with in the statistics number 9,659 patients. The tables deal only with the vaccinated cases, not with the unvaccinated, and are arranged to illustrate such points as: (1) the age incidence and relative fatality of scar-bearing vaccinated cases; (2) the classification of vaccinated cases according to the character of the scars; (3) the proportional case-mortality among scar-bearing cases arranged with respect to age periods and character of scars; (4) the comparison of the case-mortality among cases showing plain scars with that of cases showing foveated scars. The conclusions arrived at are: (1) That scars combining the qualities of area, number, and foveation are associated with a lower mortality at all ages and a lower case incidence in old age than scars which are deficient in these qualities; (2) that foveation is not essential but is not negligible; (3) that number is not essential; (4) that area is not negligible. The points found incapable of proof or disproof by the figures available are: (1) that area is not essential; (2) that number is not negligible. R. W. C. Pierce [50] publishes notes of a recurrent varioloid rash following vaccination. The patient, a schoolboy 15 years of age, was vaccinated successfully with the same lymph as that used for numerous schoolfellows on December 5th. On December 24th he developed a vesicular rash which was diagnosed as small-pox and ran the usual course of a mild case. On March 6th he again developed a vesicular rash which was diagnosed by four medical men as small-pox, but which followed a not quite normal course. The face was but little involved, febrile reaction and malaise were absent, shotty vesicles were felt in the palms. The case was, it is suggested, one of recurrent varioloid or perhaps one of generalised vaccinia, and is recorded as possibly throwing some light on the relation between variola and vaccinia. Can vaccinia, the recorder suggests, partly revert to its original character in a highly susceptible subject? A. B. Green [51] calls attention to the fact that a one-half per cent. solution of chloroform in distilled water instead of taking several weeks, as in the case of glycerinated calf lymph, to destroy all extraneous organisms, will do so in from one to six hours. He points out several advantages to be thus obtained by the substitution of chloroform solution for glycerine in the sterilisation of vaccine such as the rapidity of production and the greater activity of the lymph so prepared which can be used by this method almost straight from the calf without having to deteriorate in strength during the sterilising period.

**Diphtheria.**—Marfan [52] discussing the diagnosis of diphtheria, concludes, as a result of very careful investigations carried on by himself, that disagreement between the clinical and bacteriological findings is rarer than is sometimes stated. He thinks that as a rule the clinical characters are sufficient for determining the diagnosis of diphtheria, from which it follows that the first injection of serum may usually be given without waiting for the bacteriological diagnosis. Referring to the diagnosis of diphtheria from the false membrane not uncommonly met with in scarlet fever he quotes the opinion of Wurtz and Bourges to the effect that the pseudo-membrane found early in scarlet fever is rarely diphtheritic while that found later on is commonly so. The opinions of other observers are, however, divided. In the case of herpetic sore throat only seen after the lesions have become confluent diagnosis may be very difficult. A sudden onset with high fever, marked headache, lassitude, and even delirium favours herpes. It must, however, be remembered that herpetic eruptions elsewhere are occasionally seen in diphtheria. Marfan thinks pultaceous secretions on the tonsils are never diphtheritic. On the other hand he finds that a true clinical follicular tonsillitis may be due to Löffler's bacillus. When caused in this way the exudation is usually found to spread over the tonsil, but he advises that serum should be injected without waiting to see if this will happen if diphtheria is epidemic, if glandular infiltration is marked, or if there is co-existing laryngitis or coryza. F. Foord Caiger [53] holds the view that severe or fatal cases of throat lesion may occur having all the clinical symptoms distinctive of diphtheria without repeated and careful examination revealing the presence of diphtheria bacilli. He also points out that virulent bacilli are occasionally found in the throats of perfectly healthy persons, and further that eminent bacteriologists differ widely as to what is and what is not a diphtheria bacillus. For practical purposes he says that a positive bacterial examination in cases of follicular exudation, or even of croup without visible faucial exudation, may be taken as conclusive, that it is wiser to ignore even two or three negative examinations in cases which from their appearance are distinctly diphtheritic in nature, but that in a doubtful case two, or, better, three, negative examinations may be accepted as evidence against diphtheria. He also points out that in a young child sore throat is usually either scarlatinal or diphtheritic, as tonsillitis, catarrhal, septic, or influenzal, is a relatively uncommon disorder in childhood. Concerning the paralysis which follows diphtheria Peter [54] states that the old idea of its being a polyneuritis only is not now generally accepted. Changes have also been found to exist in the nerve trunks and roots, the ganglia, spinal cord and brain and their membranes. He accepts the classification of Monicatide, who in 1896 suggested the following grouping:—(1) Pure muscular without nerve involvement; (2) polyneuritis; (3) spinal cord lesion, either localised in the grey matter or affecting grey and white matter; (4) cerebral hæmorrhage, chiefly due to circulatory change, and therefore, he admits, a not quite defensible group. He reports two cases—a brachial monoplegia in a boy of 2 and a hemiplegia in a boy of 5—probably to be placed in the fourth group. Aubertin [55] finds no constant connection now between the part of the body attacked by false membrane and the mode of appearance of the paralysis. Paralysis is more frequent in adults, either because cases in adults are more severe, or because of their more tardy admission to hospital.

[43] Med. Rec., July 4, p. 24. [44] Ibid, Aug. 1, p. 183. [45] Lancet, July 18, p. 174. [46] Brit. Med. Jour., July 25, p. 219. [47] Lancet, Sept. 19, p. 851. [48] Ibid, Sept. 12, p. 753. [49] Ibid, Aug. 18 and Aug. 22, p. 549. [50] Ibid., Aug. 1, p. 305. [51] Med. Rec., July 4, p. 29. [52] Med. Chron., July, p. 234. [53] Ibid. [54] Ibid. [55] Ibid.

